# Stability and Biological Activity Evaluation of Chlorantraniliprole Solid Nanodispersions Prepared by High Pressure Homogenization

**DOI:** 10.1371/journal.pone.0160877

**Published:** 2016-08-08

**Authors:** Bo Cui, Lei Feng, Chunxin Wang, Dongsheng Yang, Manli Yu, Zhanghua Zeng, Yan Wang, Changjiao Sun, Xiang Zhao, Haixin Cui

**Affiliations:** Institute of Environment and Sustainable Development in Agriculture, Chinese Academy of Agricultural Sciences, Beijing, China; Waseda University, JAPAN

## Abstract

Poorly water-soluble compounds are difficult to develop as pesticide products and face great challenges in water-based and environmentally friendly formulation development. In this study, high pressure homogenization combined with lyophilization was adopted to prepare the solid nanodispersions of chlorantraniliprole with poor solubility and high melting point. The mean particle sizes of the solid nanodispersions with different pesticide contents were all less than 75 nm, even when the content was up to 91.5%. For the 2.5% chlorantraniliprole solid nanodispersion with the mean particle size of 29 nm, the suspensibility and wetting time in water were 97.32% and 13 s, respectively. The re-dispersibility and wettability were superior to those of conventional water dispersible granules. The retention on the rice leaf of 18.7 mg/cm^2^ was 1.5 and 3 times that of commercial aqueous suspension concentrate and pure water. The bioassay result to diamondback moths indicated that the toxicity of the solid nanodispersion was 3.3 and 2.8 times that of technical and aqueous suspension concentrate, respectively. Moreover, the solid nanodispersion has the advantages of total avoidance of organic solvents, significant reduction of surfactants and feasibility of obtaining high concentration nanoformulations. The solid nanodispersion is an attractive candidate for improving pesticide solubility and efficacy, and its application in crop production will reduce both residues in food and environmental pollution of pesticide.

## 1 Introduction

Pesticide as an important kind of agrochemicals has been widely applied in the world for plant protection and decreasing production loss [1]. However, most of effective pesticide compounds have poor solubility in water which limits the development of their formulations with high efficacy and safety as stated in our previous article [1]. The conventional pesticide formulations like wettable powder (WP) and emulsifiable concentrate (EC) have disadvantages of dust drift, overuse of organic solvent and low efficacy, which lead to expensive cost and environmental pollution [2–4]. Recently, nanotechnology has been exploited to solve the above problems. According to the Ostwald-Freundlich and Noyes-Whitney equations, the saturation solubility and dissolution rate increase with decreasing particle size [5–7]. In addition, the larger surface area of nanoparticles can also improve the coverage, adhesion and penetration of particles to the surface of crop leaves and targeted organisms, and further enhance the bioavailability of pesticide as described in other literatures [1,8–11].

The main techniques for producing nanoformulations involve the top-down method such as wet-milling and high pressure homogenization (HPH) [12,13], and the bottom-up method like microprecipitation and supercritical fluid [1,14,15]. Compared to the bottom-up technology, almost any compound with poor solubility can be processed with the top-down process, despite of being poorly soluble in aqueous and simultaneously in non-aqueous media [16]. The HPH process refers to passing a coarse dispersion into a vessel through a very small homogenization gap. High velocity and pressure induce shear force, cavitation force and particle collision to break big particles into small ones [17,18]. The advantages of HPH technology are free of organic solvent, ease of scale up and avoidance of contamination with erosion from milling pearls during the wet-milling process. This technology has been extensively used in the food, pharmaceutical and biotechnology industries [19]. Nevertheless, relevant reports in the field of agriculture are rare.

The nanoformulation of pesticide has attracted extensive attention and the corresponding formulations including microemulsion, nanoemulsion and nanosuspension have been explored [20,21]. However, the above formulations are all liquid form and have problems of stability for the nanosuspension and heavy use of surfactant for the self-emulsifying systems. A solid nanodispersion is a nanoformulation with hydrophobic pesticide compound dispersed in a solid hydrophilic matrix as described in detail previously [1]. It can not only maintain the desirable solubility and dispersibility of water-based nanoformulations, but also decrease surfactant content and improve the stability and safety during storage and transportation.

**Fig 1:**
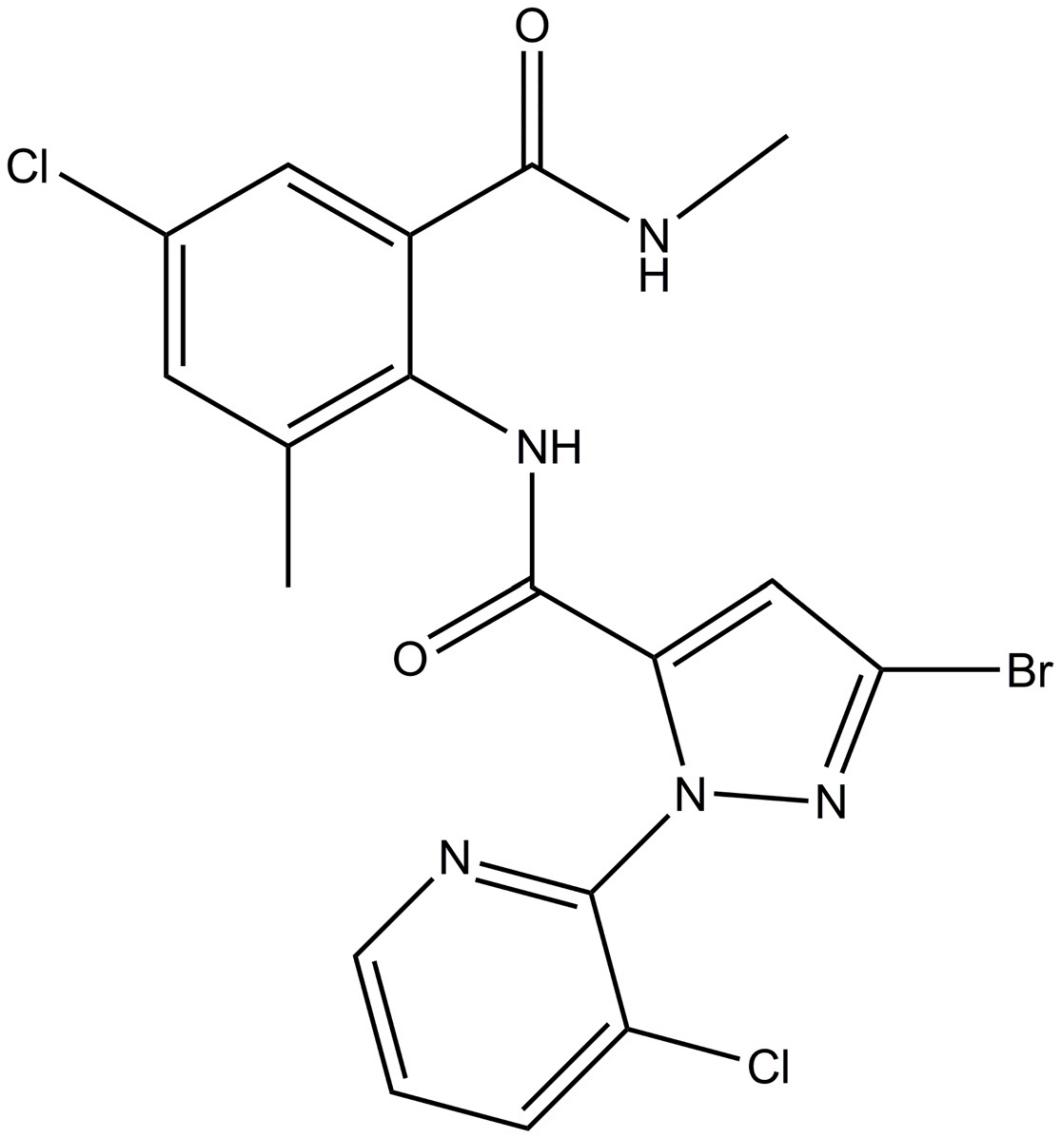
Chemical structure of chlorantraniliprole.

Chlorantraniliprole, [3-bromo-N-[4-chloro-2-methyl-6-[(methyl amino) carbonyl] phenyl]-1-(3-chloro-2-pyridinyl)-1H-pyrazole-5-carboxamide], is a new anthranilic diamide pesticide developed by DuPont Crop Protection (Fig 1) [22]. As described previously, it has a novel mode of action called ryanodine receptor activators which can lead to the depletion of intracellular calcium stores and cause impaired muscle regulation, paralysis and insect death [22,23]. The pesticide exhibits remarkable insecticidal efficacy and mammalian safety, and has been widely used for pest control in agriculture [24–26]. The current dominant formulations for chlorantraniliprole are aqueous suspension concentrate (SC) and water dispersible granule (WG). The construction of the chlorantraniliprole solid nanodispersion may further improve the pesticide stability, bioavailability and transportation convenience.

In our previous work, a solid nanodispersion of lambda-cyhalothrin was developed based on melt-emulsification and high-speed shearing methods which were suitable for pesticide compounds with melting points lower than 100°C [1]. In this present investigation, high pressure homogenization combined with lyophilization was successfully applied to prepare the solid nanodispersions of chlorantraniliprole with poor solubility and high melting point (208–210°C). This method is applicable for poorly water-soluble pesticides regardless of their melting points. The chlorantraniliprole solid nanodispersion was characterized with respect to crystallinity, suspensibility, wettability, retention, stability and biological activity. This solvent-free nanoformulation substantially reduced the surfactant content relative to conventional formulations and enhanced the safety and environmental friendliness of pesticide. In addition, the high concentration nanoformulation with 91.5% pesticide could be obtained using this method and has important application prospect for ultra-low volume and aerial spray.

## 2. Materials and Methods

### 2.1. Materials

Chlorantraniliprole (96%) and standard hard water (ρ(Ca^2+^ + Mg^2+^) = 342 mg/l) were obtained from China Agricultural University. 1-Dodecanesulfonic acid sodium salt (SDS, 99%, CAS number: 2386-53-0), sodium ligninsulfonate (SL, CAS number: 8061-51-6), polyvinylpyrrolidone K90 (PVP K90, 90%, CAS number: 9003-39-8, average molecular weight: 360000), polyethylene glycol mono-4-nonylphenyl ether (PGME, n≈15, CAS number: 26027-38-3), hexadecyltrimethylammonium bromide (CTAB, 98%, CAS number: 57-09-0) and sucrose (99%, CAS number: 57-50-1) were purchased from J&K Scientific Ltd. (Beijing, China). Hydroxypropyl methylcellulose (HPMC, CAS number: 9004-65-3, viscosity: 2600–5600 cP) was obtained from Sigma-Aldrich Shanghai Trading Co., Ltd. (Shanghai, China). Maleic rosin-polyoxypropylene-polyoxyethylene ether sulfonate (MRES, SP-SC29, number-average molecular weight: 1300) and polycarboxylate (SP-2728, number-average molecular weight: 13200) were provided by Sinvochem S&D Co., Ltd. (Jiangsu, China). The chlorantraniliprole SC (200 g/l, CORAGEN) and WGs (35%, ALTACOR and 35%, JIATENG) were purchased from DuPont Agricultural Chemicals Ltd. (Shanghai, China). The chlorantraniliprole granule (GR, 0.4%, FERTERRA) was bought from Sinon Chemical (China) Co., Ltd. (Shanghai, China). All the chemicals were used as received.

### 2.2. Preparation of the Chlorantraniliprole Solid Nanodispersions

The preparation processes of the solid nanodispersions with 2.5%, 7.5%, 22.5% and 67.5% chlorantraniliprole were almost exactly the same except for the adding amount of sucrose. Taken the 2.5% chlorantraniliprole solid nanodispersion as an example, the detailed preparation procedure was as follows. Firstly, 0.076 g MRES and 0.076 g polycarboxylate were dissolved in 58 ml water to get a colorless and transparent solution by stirring at 800 rpm for 5 minutes on a magnetic stirrer (RCT Basic, IKA^®^-Works Guangzhou, Guangzhou, China). After adding 3.166 g chlorantraniliprole into the above solution, the mixture was further stirred at 800 rpm for 5 minutes by a magnetic stirrer (RCT Basic, IKA^®^-Works Guangzhou, Guangzhou, China) and emulsified at 10000 rpm for 15 minutes by a shearing machine (C25, ATS Engineering Ltd., Vancouver, Canada) to make the pesticide particles uniformly suspend in the suspension. Secondly, the prepared suspension was subjected to a high pressure homogenizer (AH-100D, ATS Engineering Ltd., Vancouver, Canada) and homogenized at 300 bar, 600 bar, 900 bar, 1200 bar and 900 bar for 10 cycles at each pressure to obtain a chlorantraniliprole nanosuspension. Here, a cycle meant that the entire aqueous dispersion passed through the homogenizer once. This definition was the same as that in other literatures [27,28]. During stirring at 800 rpm on a magnetic stirrer (RCT Basic, IKA^®^-Works Guangzhou, Guangzhou, China), 118.242 g sucrose was added slowly. Then water was removed using a freeze drier (FD-81, EYELA, Tokyo, Japan) to obtain the 2.5% chlorantraniliprole solid nanodispersion.

The difference between the preparation of the 91.5% chlorantraniliprole solid nanodispersion and the above procedure was that sucrose was not added in the process. Firstly, the nanosuspension with 0.076 g MRES, 0.076 g polycarboxylate and 3.166 g chlorantraniliprole was produced according to the above method. Then the aqueous nanosuspension was directly lyophilized by a freeze drier (FD-81, EYELA, Tokyo, Japan) to acquire the solid nanodispersion with 91.5% pesticide.

### 2.3. Particle Size and Zeta Potential Measurements

The particle size, polydispersity index (PDI) and zeta potential of the samples were characterized at 25°C using a Zetasizer Nano ZS 90 (Malvern, Worcestershire, UK). Particle size measured by dynamic light scattering (DLS) was expressed by the mean size and 90% diameter percentile (D90). Each sample was measured in triplicate. All sizes and PDIs were recorded as mean ± standard deviation (S.D.).

### 2.4. Morphological and Structural Characterizations of the Nanoparticles

The morphological characterizations of the nanoparticles were performed using a scanning electron microscope (SEM) and a transmission electron microscope (TEM). SEM imaging was conducted by a scanning electron microscope (JSM-7401F, JEOL, Tokyo, Japan) at 3 kV. 3 μl of the aqueous dispersion with re-dispersed nanoparticles was dropped on a freshly cleaned silicon slice. The sample was air-dried and coated with platinum (thickness ≤ 2 nm) by a sputter coater (Beijing Elaborate Technology Development Ltd., Beijing, China) using an electric current of 2 mA for 3 minutes. The images were recorded at low electron image (LEI) mode and the work distance (WD) was 8.1 mm.

The morphology of the nanoparticles was also visualized by a transmission electron microscope (HT7700, HITACHI, Tokyo, Japan). 3 μl of the sample dispersion was dropped on a copper grid. The grid was left overnight for complete dryness before TEM imaging at 80 kV and 10 μA.

X-ray diffraction (XRD) was applied to evaluate the crystallinity of the samples by a diffractometer (D8 ADVANCE, Bruker AXS Inc., Karlsruhe, Germany) using CuKα radiation. The measurement conditions were as follows: tube voltage of 40 kV, tube current of 40 mA, step scan mode with a step size of 2θ = 0.02°, and counting time of 0.1 s per step.

### 2.5. Determination of Chlorantraniliprole Content

The content of the active ingredient was determined by high performance liquid chromatography (HPLC) (WAT035876, Waters Co., Milford, MA, USA) using a C18 column (5 um, 4.6 mm*250 mm, Shiseido, Tokyo, Japan) at room temperature. The mobile phase was composed of acetonitrile and water (60:40). The flow rate was 1.0 ml/min, and the UV detector wavelength was 254 nm. The Milli-Q water (18.2 MΩ.cm, TOC ≤ 4 ppb) was used for the preparation of all the solutions in this measurement.

### 2.6. Suspensibility Test

The suspensibility was tested by the similar method taken in previous study [1]. The 10.4167 g powder of the 2.5% chlorantraniliprole solid nanodispersion was added slowly to a beaker containing 50 ml standard hard water (30 ± 1°C) and swirled by hand in a circular motion at a rate of about 120 times per minute for 2 minutes. The suspension was transferred to a 250-ml measuring cylinder after placing it in a water bath at 30 ± 1°C for 13 minutes. Then 200 ml standard hard water was used to rinse the beaker and fill the cylinder to scale. Subsequently, the measuring cylinder was stoppered and inverted 30 times by hand, and kept standing in the 30 ± 1°C water bath for 30 minutes. After removing the top 225 ml of the dispersion, the pesticide contents of the original suspension and the remaining 25 ml of the dispersion were measured by HPLC. In the control experiments, 0.7163 g WGs (ALTACOR and JIATENG) and 1.2563 g SC were weighed and the measurements followed the same steps as described above.

### 2.7. Wettability Test

The 100 ml standard hard water was added into a 250-ml beaker which was placed in a water bath at 25 ± 1°C. When the temperature of the standard hard water reached 25°C, 5.000 g sample was poured on the water surface at once. Immediately, time was recorded with a stopwatch until the powder was entirely wetted by water. The average value of three tests was adopted. The 2.5% chlorantraniliprole solid nanodispersion, the commercial WGs (ALTACOR and JIATENG) and GR were all tested under the same condition.

### 2.8. Retention Test

Firstly, the 2.5% chlorantraniliprole solid nanodispersion and commercial SC were diluted into aqueous dispersions containing 0.015 mg/ml active ingredient. Then the weight of each rice (*Oryza sativa* L.) leaf was weighed using an electronic balance (ME204E, METTLER TOLEDO, Zurich, Switzerland) and its area was measured by a leaf area meter (Yaxin-1241, Beijing Yaxin Science Instrument Technology Co., Ltd., Beijing, China). Afterwards, the leaves were fully immersed in the above dispersions and pure water which was as a control test. After 10 s, each leaf was pulled out when there were no droplets falling from the surface. Finally, it was placed in a weighed culture dish to get the weight of the leaf after immersion. The weights and areas of the leaves were accurate to 0.1 mg and 0.1 cm^2^, respectively. The average value of three tests was adopted.

### 2.9. Bioassays

Bioassays were performed using the leaf-dip method as described in the research of the lambda-cyhalothrin solid nanodispersion [1]. In the biological activity assay of the chlorantraniliprole technical (TC), the TC powder was added to dimethyl sulfoxide and treated with an ultrasonic machine (KQ-500DE, Kunshan Ultrasonic Instruments Co., Ltd., Jiangsu, China) for 5 minutes to acquire a 10 g/l solution. Then it was diluted with Triton X-100 aqueous solution to obtain the dispersions with different chlorantraniliprole concentrations and 0.05% Triton X-100. For the chlorantraniliprole solid nanodispersion and SC, the samples were directly diluted with pure water to different concentrations followed by 5 minutes of ultrasound. Subsequently, rape (*Brassica campestris* L.) leaves were immersed in the above dispersions for 10 s. Afterwards, the leaves were air-dried and placed in a culture dish with a filter paper. Ten second-instar diamondback moth (*Plutella xylostella* L.) larvae were introduced into each dish, and three replications were carried out. The control test in which leaves were only treated with 0.05% Triton X-100 solution was also conducted for comparison. Mortality was assessed after treatment for 48 h. Concentration–mortality data were analyzed using DPS 8.1 (Refine Information Technology Co., Ltd., Hangzhou, China).

### 2.10. Statistical Analysis

The data were analyzed by one-way analysis of variance (ANOVA) and Duncan’s multiple range test. Statistical analysis was performed with the software package SPSS and a value of P < 0.05 was deemed to be statistically significant.

## 3 Results and Discussion

The preparation of the chlorantraniliprole solid nanodispersion involved two sequential processes: producing the chlorantraniliprole nanosuspension by HPH and solidifying the aqueous dispersion by adding water-soluble carrier and removing water. As demonstrated previously [1], the particle size and distribution of the pre-prepared nanosuspension have a crucial impact on the properties of the final solid nanodispersion, thus the composition and preparation parameters of the nanosuspension have been investigated in detail using the particle size and PDI as evaluation indices.

### 3.1. Surfactant Screening and Composition Optimization

Surfactant as an indispensable part of pesticide formulation remarkably affects the formulation performance. In this research, four anionic (SDS, SL, MRES and polycarboxylate), three nonionic (PVP K90, HPMC and PGME) and one cationic (CTAB) surfactants were compared. The nanosuspensions containing 1% (w/w) chlorantraniliprole and 0.2% (w/w) single surfactant were produced by homogenization at 300 bar, 600 bar, 900 bar, 1200 bar and 900 bar for 10 cycles at each pressure. The pesticide particles suspended in the dispersions through the stabilizing effect of the surfactants. The amphiphilic surfactants adsorbed on the pesticide surface by hydrophobic interactions while leaving the hydrophilic end stretching outside. This structure could enhance the wettability and dispersibility of the poorly water-soluble pesticide in water. Table 1 shows the surfactant effect on the particle size and dispersibility of the nanosuspensions. Among the eight surfactants, MRES and polycarboxylate reduced the mean size and D90 of the particles to less than 29 nm and 76 nm, respectively. The -SO3^-^ group on the MRES molecular skeleton and -COO^-^ group of polycarboxylate may interact with -NH group of chlorantraniliprole molecule through hydrogen bonds and make the surfactants adsorb on the pesticide surface [29,30]. Furthermore, the Van der Waals force between chlorantraniliprole and surfactants may also enhance their intermolecular interactions. The anionic surfactants on the pesticide surface made the particles negatively charged and repel each other to prevent the formation of large particles. Meanwhile, the hydrophobic chains of MRES and polycarboxylate could further induce steric effect against aggregation. Considering both MRES and polycarboxylate were capable of significantly reducing the mean size (P < 0.05) and distribution of the particles, the two surfactants were mixed and the appropriate proportion between them was further investigated.

**Table 1:** Effect of surfactants on the particle size and dispersibility of the chlorantraniliprole nanosuspensions.

Surfactant^a^	Mean size (nm) ± S.D.	D90^b^ (nm) ± S.D.	PDI^c^ ± S.D.^d^
SDS	35 ± 1c	105 ± 8ab	0.34 ± 0.01a
SL	34 ± 1c	86 ± 7bc	0.24 ± 0.01bc
MRES	28 ± 1d	75 ± 8cd	0.25 ± 0.03bc
Polycarboxylate	24 ± 1e	66 ± 3d	0.25 ± 0.01bc
PVP K90	49 ± 1a	117 ± 24a	0.22 ± 0.03c
HPMC	43 ± 0b	91 ± 5bc	0.25 ± 0.01bc
PGME	35 ± 1c	104 ± 3ab	0.26 ± 0.01b
CTAB	43 ± 3b	80 ± 11cd	0.17 ± 0.02d

^a^ SDS: 1-dodecanesulfonic acid sodium salt; SL: sodium ligninsulfonate; MRES: maleic rosin-polyoxypropylene-polyoxyethylene ether sulfonate; PVP K90: polyvinylpyrrolidone K90; HPMC: hydroxypropyl methylcellulose; PGME: polyethylene glycol mono-4-nonylphenyl ether; CTAB: hexadecyltrimethylammonium bromide.

^b^ D90: particle size expressed by the 90% diameter percentile.

^c^ PDI: polydispersity index.

^d^ S.D.: standard deviation of three measurements.

Different letters at each data indicate significant differences according to Duncan’s multiple range test at P < 0.05.

Using MRES and polycarboxylate as composite surfactants, the particle sizes and PDIs of the chlorantraniliprole nanosuspensions with different ratios of MRES to polycarboxylate were shown in Table 2. In contrast with the nanosuspensions stabilized by single surfactant, the mean size and D90 of the nanosuspension containing composite surfactants decreased even reducing the relative content of surfactant to pesticide. While the two surfactants adsorbing on the pesticide surface, both electrostatic repulsion and steric stabilization effects contributed to the nanosuspension stability by preventing particles from aggregation. The 1:1 (w/w) ratio of MRES to polycarboxylate was chosen in the following preparation of the chlorantraniliprole solid nanodispersions.

**Table 2:** Effect of the ratio of MRES^a^ to polycarboxylate on the particle size and dispersibility of the chlorantraniliprole nanosuspensions^b^.

Ratio of MRES to polycarboxylate (w/w)	Mean size (nm) ± S.D.	D90^c^ (nm) ± S.D.	PDI^d^ ± S.D.^e^
1:3	13 ± 1b	33 ± 3ab	0.23 ± 0.01a
1:1	13 ± 0b	30 ± 1b	0.23 ± 0.00a
3:1	14 ± 0a	36 ± 2a	0.23 ± 0.00a

^a^ MRES: maleic rosin-polyoxypropylene-polyoxyethylene ether sulfonate.

^b^ The nanosuspensions containing 5% (w/w) chlorantraniliprole and 0.25% (w/w) surfactants were prepared by homogenization at 300 bar, 600 bar, 900 bar, 1200 bar and 900 bar for 10 cycles at each pressure.

^c^ D90: particle size expressed by the 90% diameter percentile.

^d^ PDI: polydispersity index.

^e^ S.D.: standard deviation of three measurements.

Different letters at each data indicate significant differences according to Duncan’s multiple range test at P < 0.05.

**Fig 2:**
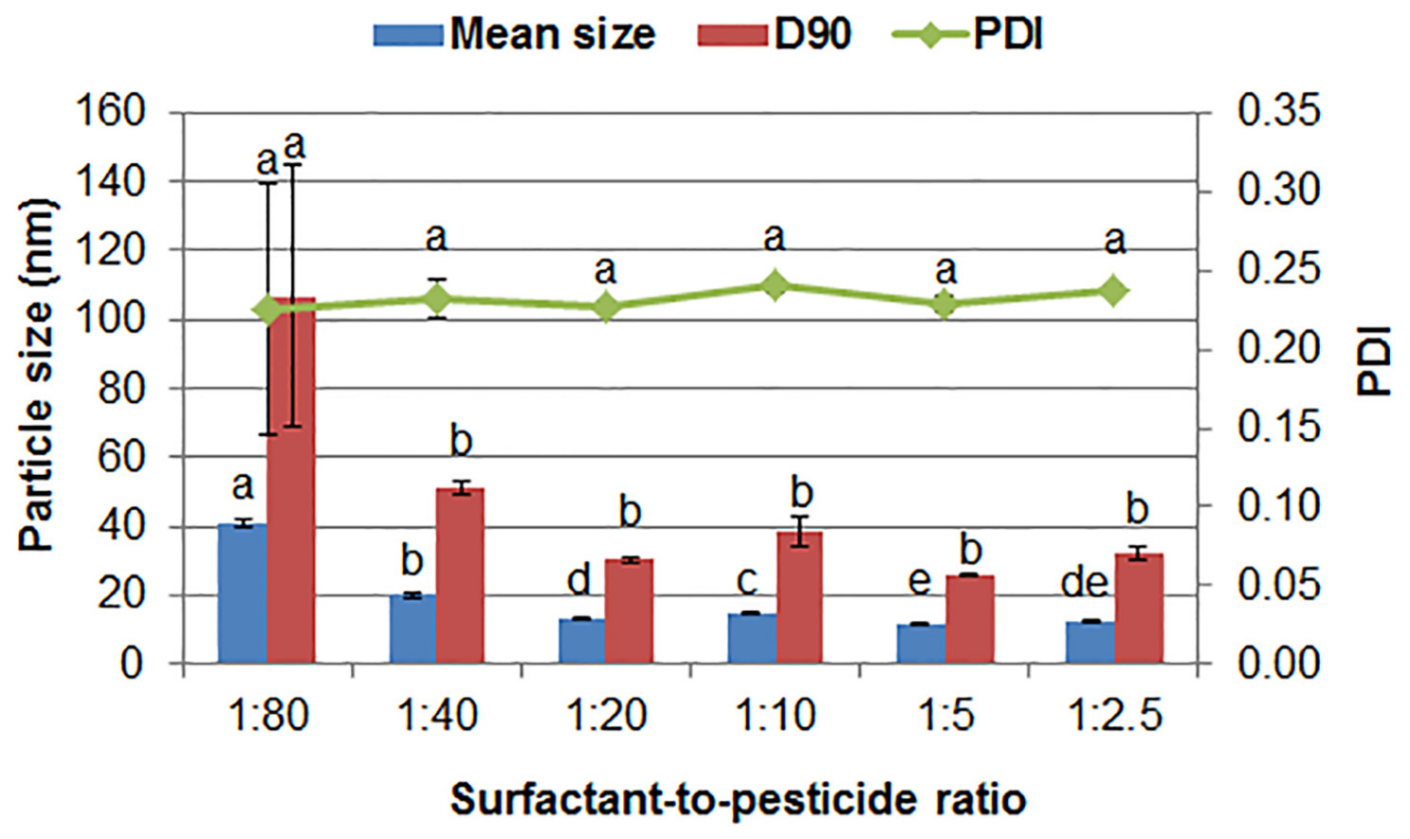
The particle size and dispersibility of the nanosuspensions containing 5% (w/w) chlorantraniliprole with different surfactant-to-pesticide ratios. D90: particle size expressed by the 90% diameter percentile; PDI: polydispersity index. Different letters at each data indicate significant differences according to Duncan’s multiple range test at P < 0.05.

The content of surfactants affects their adsorption amount on the surface of poorly water-soluble pesticide, and further influences the dispersibility and stability of the formulation. Fig 2 shows the effect of surfactant-to-pesticide ratio on the particle size and distribution of the chlorantraniliprole nanosuspensions. When the surfactant was not enough to provide efficient electrostatic repulsion and steric hindrance, the neighboring nanoparticles may approach and aggregate. Indeed, as the ratio increased from 1:80 to 1:20, the mean size and D90 of the pesticide nanoparticles decreased gradually. However, the particle size and PDI changed little and even appeared a slight increase when the ratio was larger than 1:20. The possible reason was that more surfactants made the interface layer of particles become thicker [31]. In addition, the large amount of surfactants would cause greater food safety and environmental issues [32,33]. Therefore, the 1:20 surfactant-to-pesticide ratio was determined for the preparation of the solid nanodispersions. The optimized surfactant content was much lower than that in most microemulsions and solid microemulsions [34–38].

### 3.2. Homogenization Process Effect

**Fig 3:**
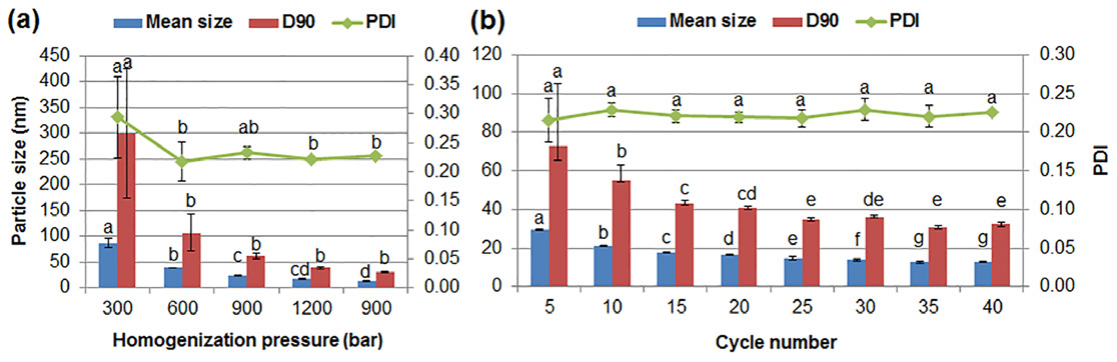
The particle size and dispersibility of the nanosuspensions containing 5% (w/w) chlorantraniliprole prepared in (a) variable pressure mode and (b) constant pressure mode. D90: particle size expressed by the 90% diameter percentile; PDI: polydispersity index. Different letters at each data indicate significant differences according to Duncan’s multiple range test at P < 0.05.

Homogenization condition has a significant impact on the particle size and structure during the preparation of nanosuspensions [18,39,40]. In this investigation, to avoid a blockage of the homogenization gap caused by large particles [41], the mixed dispersion of pesticide and surfactants was first sheared to reduce the particle size to about 600 nm before passing it into the homogenizer. The effects of both homogenization mode and pressure on the particle size and dispersibility of the chlorantraniliprole nanosuspensions have been explored. As the homogenization pressure increased, the enhanced shear and cavitation forces made big particles split into small ones, consistent with the result in Fig 3a. The reduction of the mean size of particles was obvious from 300 bar to the first 900 bar (P < 0.05), but the change became slight during the further homogenization process. It has been reported that input of more energy may lead to “over-processing” and induce particle growth because it accelerates particle movement and causes aggregation [9,42,43]. Similar phenomenon also exists in the milling process [9,44]. Therefore, in order to substantially improve the dispersibility of the nanosuspension while avoiding particle aggregation, a lower 900 bar was adopted following 1200 bar to further enhance the system stability. The above result demonstrates that homogenization pressure could effectively regulate the size and distribution of the nanoparticles. The comparative study was conducted in the constant pressure mode at 1200 bar. As shown in Fig 3b, the mean size and D90 of the particles decreased progressively with increasing cycle number. The increase of cycle number provided sufficient time for the surfactants to adsorb onto the pesticide surface and breaking large particles. By comparison, the final particle size in the variable pressure mode was equal to that after passing 35 cycles at 1200 bar. Considering the energy consumption problem, the variable pressure mode was chosen.

The water-based suspensions are thermodynamically unstable systems that tend to break down over time due to a variety of physicochemical mechanisms, for example, gravitational separation, flocculation and Ostwald ripening [45]. In order to improve the stability and prolong shelf life of the formulation, the chlorantraniliprole nanosuspensions were transformed into solid nanodispersions by lyophilization. During this process, sucrose was added before freeze-drying. Here, sucrose as water-soluble carrier can not only act as antifreeze agent to protect dispersion from freezing and desiccation impairment, but also accelerate redispersion of the solid nanodispersions as proved by other systems [1,46,47]. Furthermore, it can also improve the suspensibility and stability of the re-dispersed dispersion by increasing its viscosity [48].

**Fig 4:**
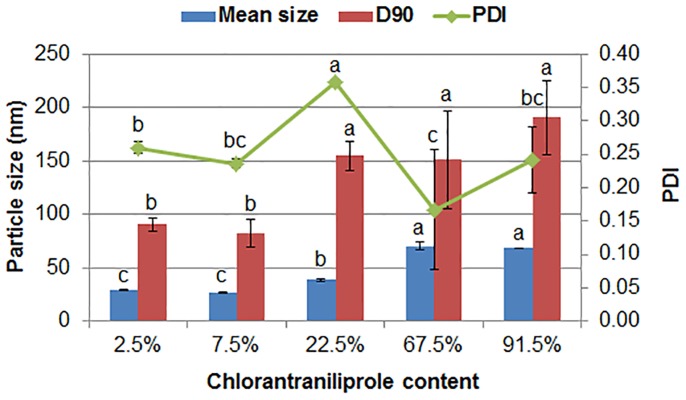
The particle size and dispersibility of five chlorantraniliprole solid nanodispersions with different pesticide contents. D90: particle size expressed by the 90% diameter percentile; PDI: polydispersity index. Different letters at each data indicate significant differences according to Duncan’s multiple range test at P < 0.05.

After getting the identical nanosuspensions by the same preparation process, the adding amount of sucrose was regulated to prepare the solid nanodispersions with different pesticide contents. The content of the active ingredient gradually increased as the proportion of sucrose in the composition decreased. For the 2.5%, 7.5%, 22.5% and 67.5% solid nanodispersions, the formulations consisted of chlorantraniliprole, composite surfactants of MRES and polycarboxylate and sucrose. By contrast, in order to maximize the pesticide content, the nanosuspension was directly lyophilized without adding sucrose to get the 91.5% solid nanodispersion. In this condition, the formulation was only composed of chlorantraniliprole and surfactants. As shown in Fig 4, the mean size of nanoparticles became larger as increasing pesticide content but all less than 75 nm. Similar phenomenon has been observed in nanosuspension systems and clarified as due to achieving a more stable state by coalescence [49]. It is noteworthy that the chlorantraniliprole content in the solid nanodispersion could reach up to 91.5%, while keeping the mean particle size, D90 and PDI at 68 nm, 191 nm and 0.24, respectively. This high concentration nanoformulation maximized the active ingredient and minimized the surfactants, solvent and carrier. It could dramatically reduce the production cost and improve safety, environmental friendliness and utilization convenience of the product. Moreover, it is an ideal candidate for oil formulations in the application of ultra-low volume and aerial spray.

### 3.3. Characterization and Evaluation of the Solid Nanodispersion

#### 3.3.1. Size and Morphology

**Fig 5:**
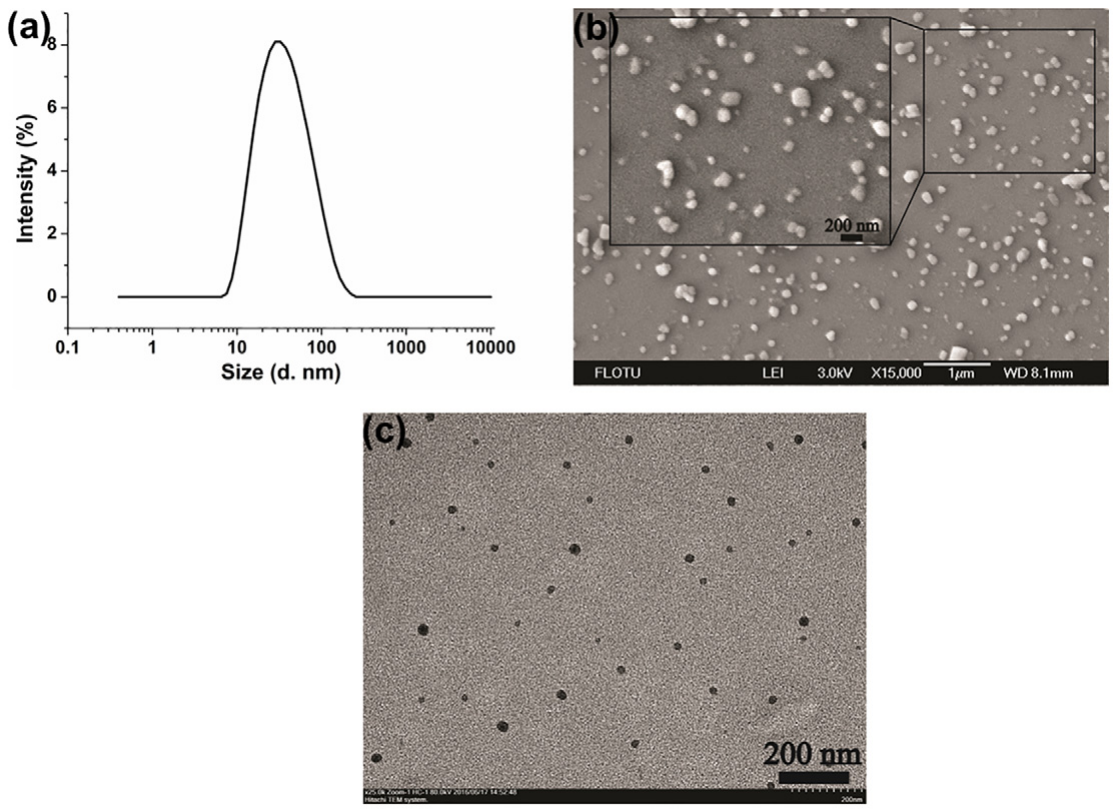
The size and morphology of the chlorantraniliprole nanoparticles. (a) Particle size measured by DLS; (b) SEM image with magnification of 15000; (c) TEM image with magnification of 25000. Size (d. nm): diameter size of the nanoparticles.

The solid nanodispersion containing 2.5% (w/w) chlorantraniliprole was taken as an example to be evaluated in detail. The mean size, D90 and PDI of the nanoparticles measured by DLS were 29 ± 1 nm, 91 ± 6 nm and 0.26 ± 0.01, respectively (Fig 5a). The slight agglomeration of particles during lyophilization has also been reported in other nanoformulations [49]. As observed from the SEM and TEM images (Fig 5b and 5c), the nanoparticle size was mainly in the range of 25 nm to 135 nm, agreed with the result of DLS. During the homogenization process, the shear, cavitation and collision forces were asymmetrically applied to the particles and resulted in the irregularity of the particle shape.

#### 3.3.2. Zeta Potential and pH

Zeta potential is a typical index to evaluate the surface charge property of particles and the physical stability of water-based formulations [50]. The zeta potential and pH of the re-dispersed solid nanodispersion were– 22 mV and 7.4, respectively. The negative zeta value demonstrates that the anionic surfactants adsorbed on the pesticide surface and made the particles present electronegativity. In general, absolute zeta potential values higher than 30 mV predict a strong long-term stability of a suspension. However, a suspension with lower zeta potential can also exhibit excellent stability when the surfactants provide steric stabilization in addition to electrostatic repulsion [27,28]. The adsorption layer of the chlorantraniliprole nanoparticles consisted of anionic polymers MRES and polycarboxylate which both acted as electrostatic repulsion and steric stabilizers. This may lead to a shift of the shear plane to a larger distance from the particle surface, and thus, to a reduction in the measured potential [28,51,52]. In addition, the pH close to neutral condition was conducive to avoiding the decomposition of active ingredient.

#### 3.3.3. Crystallinity

**Fig 6:**
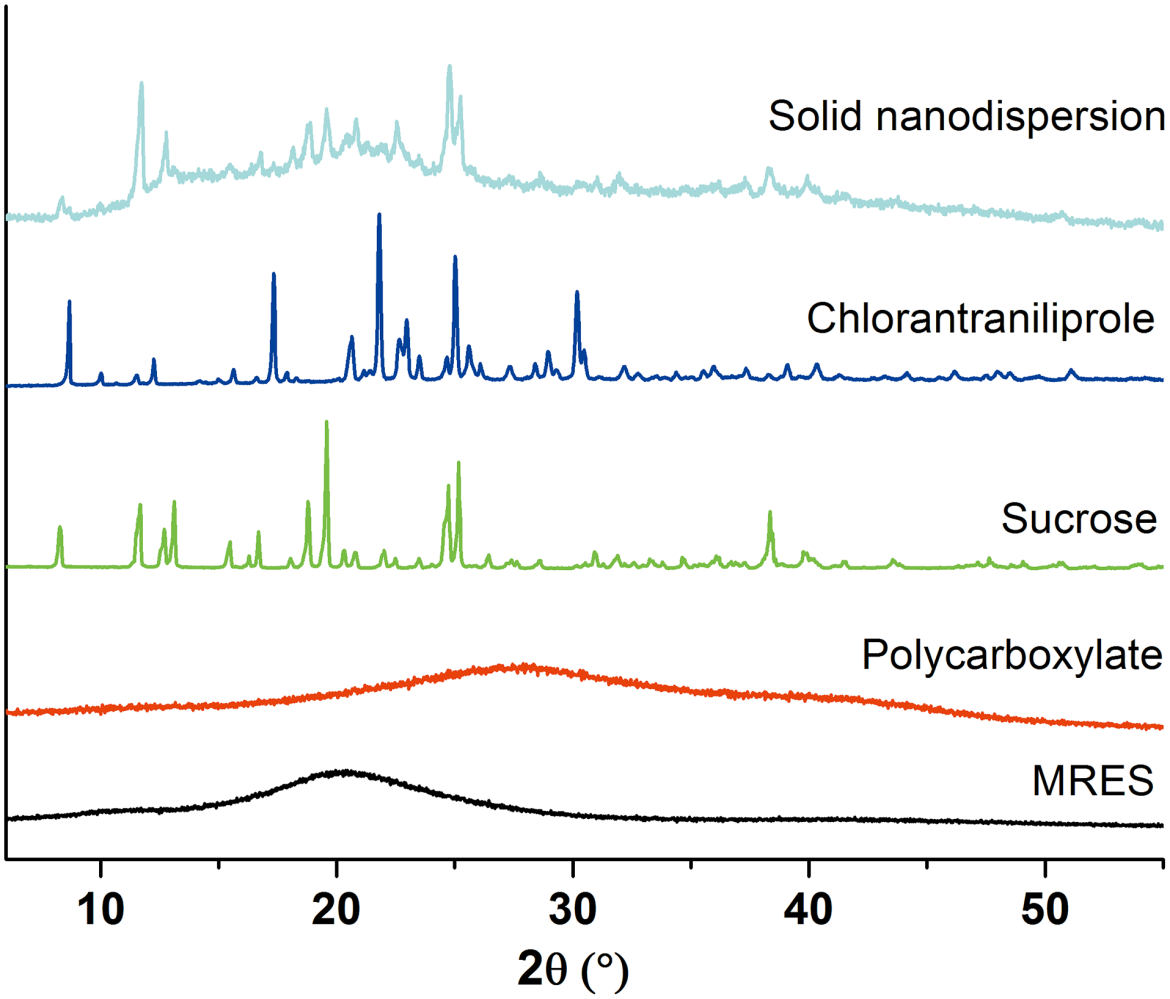
XRD patterns of the chlorantraniliprole solid nanodispersion and pure components in the formulation. MRES: maleic rosin-polyoxypropylene-polyoxyethylene ether sulfonate.

As shown in Fig 6, XRD pattern of the solid nanodispersion presented a certain degree of amorphous characteristic compared to the pure chlorantraniliprole nanocrystal, owing to the amorphous surfactants covering the pesticide surface. The intense peaks of the solid nanodispersion mainly resulted from sucrose crystal which accounted for the largest proportion in the formulation composition. As reported, HPH can induce structural change of materials [53]. The characteristic peaks of pure pesticide at 8.7°, 10.0°, 17.3° and 30.2° observed in the pattern indicates the preservation of pesticide crystal structure. The crystalline state was stable during storage and the amorphous component could promote the dissolution of poorly water-soluble compound [54–56]. The coexistence of crystalline and amorphous structures will play an important role in improving storage stability and water solubility of the chlorantraniliprole solid nanodispersion.

#### 3.3.4. Suspensibility

Suspensibility is an important indicator of the re-dispersibility of solid formulations and the kinetic stability of suspensions. The value was measured according to CIPAC MT 184 and calculated by the following equation:

$$\text{Suspensibility}(\%)=\frac{10}{9}\times \frac{{\text{m}}_{1}-{\text{m}}_{2}}{{\text{m}}_{1}}\times 100$$

Here, m_1_ (mg) and m_2_ (mg) are the pesticide contents of the original suspension and the left 25-ml dispersion at the bottom, respectively. As reported in the literatures, the suspensibilities of bacillus marinus WP and fluopicolide·pyraclostrobin WG after formulation optimization were 73.13% and 90.56% [57,58]. In this research, the suspension properties of the chlorantraniliprole solid nanodispersion and three commercial products were compared under the same condition. The suspensibilities of the chlorantraniliprole SC, ALTACOR WG and JIATENG WG were 96.63%, 93.60% and 86.72%, respectively. By contrast, the 97.32% suspensibility of the solid nanodispersion indicated that the re-dispersibility and stability of the nanoformulation were further improved. There is an inverse relationship between suspensibility and particle size [59], because the relatively small particle size means that Brownian motion may dominate the gravitational force [60]. In addition, sucrose as a thickener increased solution viscosity and decreased particle sedimentation velocity. Therefore, the excellent suspension characteristic of the solid nanodispersion could attribute to the small size effect and formulation composition.

#### 3.3.5. Wettability and Retention

Wettability and retention influence the pesticide efficacy by affecting spread and adhesion of aqueous dispersions on leaves after spraying. The wettability was evaluated according to CIPAC MT 53 and the wetting times of the commercial chlorantraniliprole GR, ALTACOR WG and JIATENG WG were 373 s, 84 s and 65 s, respectively. In contrast, the solid nanodispersion could be wetted by water within 13 s which was only one fifth of that for conventional formulations. The retention (R_m_, mg/cm^2^) was measured according to the literature [61] and calculated by the following equation:

$${\text{R}}_{\text{m}}=\frac{{\text{W}}_{1}-{\text{W}}_{0}}{\text{S}}$$

Here, W_0_ (mg) and W_1_ (mg) are the weights of the hydrophobic rice (*Oryza sativa* L.) leaf before and after immersing in aqueous dispersions, S (cm^2^) is the area of the rice (*Oryza sativa* L.) leaf. As shown in Table 3, the retention of the chlorantraniliprole solid nanodispersion was 1.5 and 3 times that of commercial SC and pure water, respectively. The above results demonstrate that the size reduction, enlarging specific surface area of particles and their contact area with leaves, is indeed beneficial to shortening wetting time and increasing retention of pesticides.

**Table 3:** The retention of the chlorantraniliprole solid nanodispersion on rice (*Oryza sativa* L.) leaves.

Formulation	Retention (mg/cm^2^)
Solid nanodispersion	18.7 ± 0.7a
SC^a^	11.1 ± 0.6b
Water	6.0 ± 0.7c

^a^ SC: aqueous suspension concentrate.

Different letters at each data indicate significant differences according to Duncan’s multiple range test at P < 0.05.

#### 3.3.6. Storage Stability

**Fig 7:**
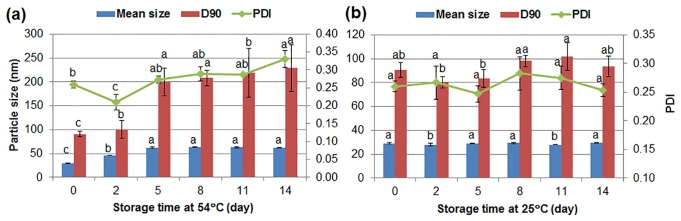
Stability of the chlorantraniliprole solid nanodispersion at (a) 54°C and (b) 25°C. D90: particle size expressed by the 90% diameter percentile; PDI: polydispersity index. Different letters at each data indicate significant differences according to Duncan’s multiple range test at P < 0.05.

The stability of the solid nanodispersion was tested according to CIPAC MT 46 and GB/T 19136–2003. As shown in Fig 7a, within five days, the mean particle size increased from 29 nm to 62 nm during storage at 54°C, but kept constant up to 14 days. The particle growth during storage also exists in microemulsions and nanosuspensions because of Ostwald ripening [12,21]. By contrast, the size and PDI of the nanoparticles remained almost unchanged during storage at 25°C, indicating excellent storage stability (Fig 7b). The amorphous characteristic of the nanoparticles caused slight aggregation at high temperature. However, the crystalline component and narrow size distribution could effectively prevent particle coalescent and recrystallization at room temperature, improving the stability of the solid nanodispersion.

#### 3.3.7. Biological Activity

The bioassay result of the chlorantraniliprole solid nanodispersion to diamondback moths (*Plutella xylostella* L.) was compared with TC and SC as shown in Table 4. The toxicity of the solid nanodispersion was 3.3 and 2.8 times that of TC and SC, respectively. It has been reported that the bioavailability of nanoemulsion is higher than that of conventional emulsion because of its small particle size and high surface-to-volume ratio [62]. Therefore, the enhanced biological activity of the solid nanodispersion here can be attributed to the improvement of the formulation performance in dispersibility, wettability and retention caused by small size effect. The formulation with high bioavailability could substantially reduce usage, decrease residue and improve environmental friendliness of pesticide.

**Table 4:** Bioassay results of three chlorantraniliprole formulations.

Formulation	Toxicity regression equation	Correlation coefficient	LC 50^a^ (μg/mL)	95% confidence limit
TC^b^	Y = 3.9289 + 1.4758x	0.9762	5.32	3.51~8.05
SC^c^	Y = 3.3843 + 2.4387x	0.9581	4.60	2.21~9.54
Solid Nanodispersion	Y = 4.6368 + 1.7317x	0.9860	1.62	1.10~2.40

^a^ LC 50: median lethal concentration.

^b^ TC: technical.

^c^ SC: aqueous suspension concentrate.

## 4 Conclusion

In this research, the solid nanodispersions of poorly water-soluble chlorantraniliprole with high melting point were prepared by high pressure homogenization combined with lyophilization. The formulation avoided organic solvent, substantially reduced surfactant amount and could increase pesticide content to 91.5%. The solid nanodispersion with mean particle size of 29 nm presented improved formulation characteristics in dispersibility, wettability, stability and bioavailability compared to the conventional pesticide formulations. Therefore, the application of the solid nanodispersion in crop protection has great potential for reducing residues in food and environmental pollution of pesticide.
